# Retraction: SETDB1 Is Involved in Postembryonic DNA Methylation and Gene Silencing in *Drosophila*

**DOI:** 10.1371/journal.pone.0194869

**Published:** 2018-03-20

**Authors:** The PLOS ONE Editors

The Office of Research Integrity (ORI) within the United States Department of Health and Human Services has completed an investigation involving this article [1] and concluded there was evidence of misconduct involving Figure 1B. As detailed in the ORI report [2], the image was used in grant application figures and two other publications [3,4], in which the same data was reported as representing different experiments.

In addition, concerns have been raised about other figures in the *PLOS ONE* article:

- There were vertical lines and background differences in Figure 4A suggestive of splicing around the Antp and CG2316 bands of dSETDB1.
- Analysis of color adjusted images revealed vertical lines suggestive of splicing in Figure 5D (pspGI for PDE, between lanes 1/2, 2/3, 3/4) and Figure 6C (5mc, after lane 2)
- Similarities were noted between Figure 4A (“S2 cell” and “Control” bands of CG2316, lanes 1, 2) and Figure 6C (“+mock RNAi” and “+Dnmt2-RNAi” of Su(var)205; lanes 2, 3)
- Similarities were noted between Figure 4A (CG2316 panel, lanes 4, 5) and Figure 6C((me3) H3-K9, lanes 6, 5)

FS asserted that the wrong image was used in error for Figure 1B in the *PLOS ONE* publication. The authors did not respond to the journal’s queries about the concerns raised regarding the other images, or our requests for the raw data underlying the figures in question.

In light of the above concerns about the integrity of the published data, the *PLOS ONE* Editors retract this article.

EK, FMB agree with the retraction. DG, MR, SS, HEB, PT, FS could not be reached.

## References

[1] Gou D, Rubalcava M, Sauer S, Mora-Bermúdez F, Erdjument-Bromage H, Tempst P, et al (2010) SETDB1 Is Involved in Postembryonic DNA Methylation and Gene Silencing in *Drosophila*. PLoS ONE 5(5): e10581 https://doi.org/10.1371/journal.pone.0010581 20498723 10.1371/journal.pone.0010581 PMC2871795

[2] https://ori.hhs.gov/content/case-summary-sauer-frank.

[3] Beisel C, Imhof A, Greene J, Kremmer E, Sauer F (2002). Histone methylation by the *Drosophila* epigenetic transcriptional regulator Ash1. Nature 419, 857–862. doi: 10.1038/nature01126 (retracted: https://www.nature.com/articles/nature14421) 12397363 10.1038/nature01126

[4] Maile T, Kwoczynski S, Katzenberger RJ, Wassarman DA, Sauer F (2004). TAF1 Activates Transcription by Phosphorylation of Serine 33 in Histone H2B. Science 304 (5673), 1010–1014. doi: 10.1126/science.1095001 (retracted: http://science.sciencemag.org/content/344/6187/981.1) 15143281 10.1126/science.1095001

